# Myoepithelial Carcinoma of the Floor of the Mouth: A Rare Salivary Gland Tumor in an Unusual Location

**DOI:** 10.7759/cureus.12321

**Published:** 2020-12-27

**Authors:** Javaria Ali, Shahzeb Munawar, Rimsha Haider, Ahmad Nawaz Ahmad, Atif A Hashmi

**Affiliations:** 1 Pathology, Liaquat National Hospital and Medical College, Karachi, PAK; 2 Internal Medicine, Liaquat College of Medicine and Dentistry, Karachi, PAK; 3 Internal Medicine, Liaquat National Hospital and Medical College, Karachi, PAK; 4 Emergency Medicine, National Institute of Blood Disease & Bone Marrow Transplantation, Karachi, PAK; 5 Otolaryngology, Liaquat National Hospital and Medical College, Karachi, PAK

**Keywords:** myoepithelial carcinoma, myoepithelial neoplasm, salivary gland tumor, floor of the mouth

## Abstract

Myoepithelial carcinomas are rare malignant salivary gland tumours encountered most commonly in the parotid gland and are amenable to surgical resection. However, when they occur at complex anatomical locations, complete resection becomes difficult due to their locally aggressive nature. Here we describe a case of a large myoepithelial carcinoma arising in the floor of the mouth and involving major structures at the skull base.

A 30-year-old female presented with a slow-growing mass in the oral cavity. Computed tomography (CT) scan showed a heterogeneous appearing lesion in the mouth floor measuring 6.7 x 5.8 x 7.3 cm. Superiorly, the lesion was extending up to the skull base, laterally extending up to the parotid gland and inferiorly up to the submandibular gland. This lesion was also encasing the internal carotid artery. Incisional biopsy under local anaesthesia was performed, and the specimen was sent for histopathological analysis. Microscopic examination showed a neoplastic lesion composed of sheets of cells with oval nuclei and clear cytoplasm with a myxoid background. Immunohistochemical expression of pan-cytokeratin (CKAE1/AE3), p63, anti-smooth muscle actin (ASMA) and glial fibrillary acidic protein (GFAP) supported the diagnosis of myoepithelial neoplasm. The patient then underwent excision of the mass followed by histological analysis, which further showed microscopic evidence of infiltration into the surrounding tissue along with areas of atypia and significant mitoses. These morphological findings supported the diagnosis of myoepithelial carcinoma. The excised tumour was reaching up to the excision margin.

Myoepithelial carcinomas are rare malignant tumours with diverse histomorphological patterns frequently present as a diagnostic challenge. The mainstay of treatment is complete surgical excision with disease-free margins, which can be challenging due to local aggressiveness and large size of these tumours. When these tumours occur in complex anatomical locations, complete excision becomes difficult, resulting in a dismal prognosis.

## Introduction

Myoepithelial carcinomas are rare salivary gland neoplasms that exclusively constitute cells with myoepithelial differentiation [1]. They predominantly affect the parotid gland with a few cases arising in the minor salivary glands of the oral cavity and palate [2]. They are slowly growing and asymptomatic tumours, presenting commonly in the fourth decade of life with a slight female predilection [3].

Grossly, they appear as unencapsulated masses with a grey to tan white cut-surface. These tumours display a diversity of morphological features and can display spindle, plasmacytoid, epithelioid, and clear cell features pushing to infiltrative borders. The stroma is typically myxoid or hyalinized with occasional pseudocyst formation.

Generally, the mean size for these tumours is 4.7cm. However, they can attain diameters of as much as 40cm and involve major structures that pose a challenge for complete resection [4]. Here we describe a case of large myoepithelial carcinoma arising in the mouth floor and involving major structures at the skull base.

## Case presentation

A 30-year-old female patient presented with a 6-month history of painless swelling in the mouth that gradually increased in size. Upon clinical examination, a large mass was seen in the oral cavity, having a smooth external appearance and filling the oral cavity and grossly displacing the tongue.

**Figure 1:**
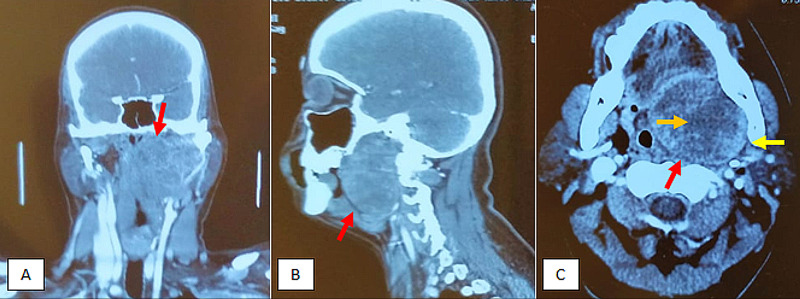
Computed tomography (CT) scan neck with contrast. (A): Coronal view of the CT scan depicting a mass in the parapharyngeal space that is superiorly abutting the base of the skull (red arrow). (B): Sagittal view of CT scan revealing the extension of the mass into the floor of the mouth (red arrow). (C): Axial view of the CT scan showing the involvement of the medial pterygoid muscle (yellow arrow), and extension of the mass into the prevertebral region (red arrow). There is also an area of internal necrosis (orange arrow).

A computed tomography (CT) scan was done, which showed a large heterogeneously appearing lesion in the floor of mouth involving the left parapharyngeal and retropharyngeal space measuring 6.7 x 5.8 x 7.3cm. Superiorly, the lesion was extending up to the skull base, laterally extending up to the parotid gland and inferiorly up to the submandibular gland. This lesion was also encasing the internal carotid artery and involving the medial pterygoid muscle (Figure 1).

**Figure 2:**
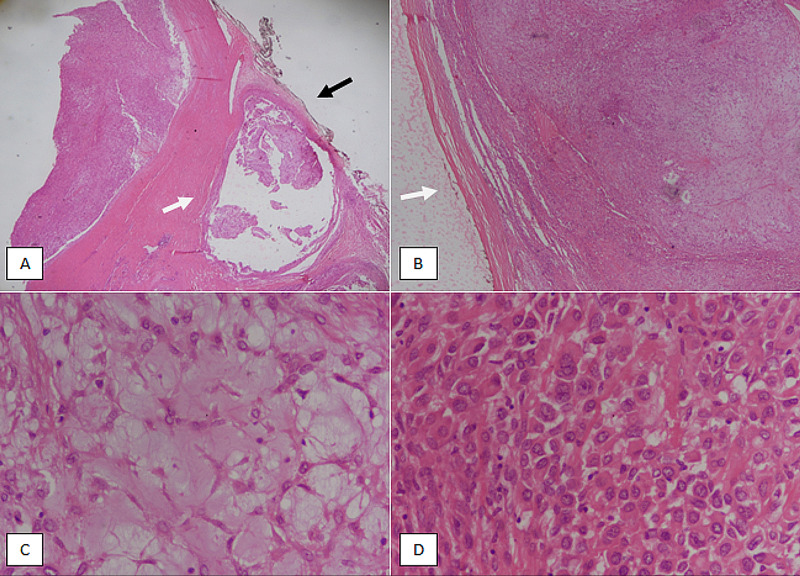
Microscopic features of myoepithelial carcinoma. (A): H & E-stained section at 20x magnification showing a tumor nodule invading into the surrounding tissue (white arrow). The tumor is also close (<1mm) from the resection margin (black arrow). (B): H & E-stained section at 40x magnification revealing circumscribed pushing borders in this area of the tumor (white arrow). (C): H & E-stained section at 400x magnification depicting myxoid background in this area of the tumor. (D): H & E-stained section at 400x magnification showing cellular area of the tumor with pronounced nuclear atypia. H & E: hematoxylin and eosin

Excision of the mass was done. Peroperatively, the mass was largely circumscribed, but at places adherent to surrounding structures. The excised specimen was sent for histological analysis. It consisted of a single unencapsulated nodular mass measuring approximately 8 x 7 x 6 cm. Microscopic examination showed a neoplastic lesion composed of solid sheets of cells with myxoid and hyalinized stroma with the occasional necrosis areas. Tumour cells had moderate nuclear atypia along with a few mitoses. The periphery of the lesion had pushing borders with focal areas of infiltration. The tumour was focally reaching up to the excision margin (Figure 2).

**Figure 3:**
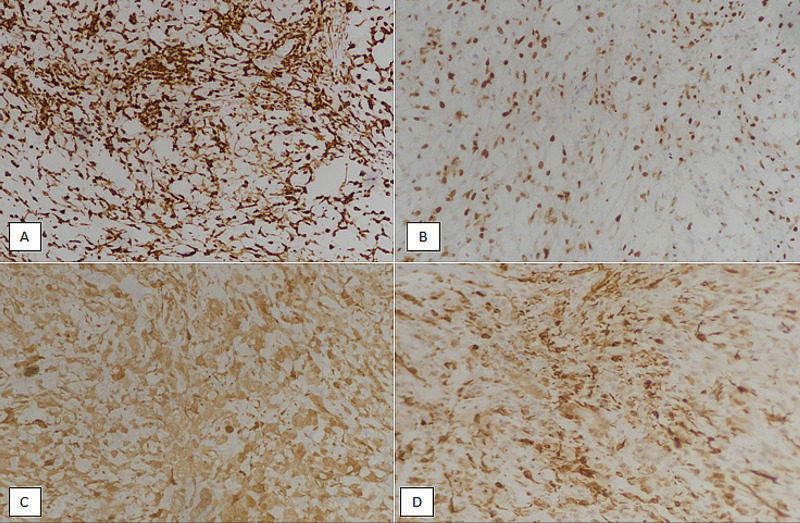
Immunohistochemical staining in myoepithelial carcinoma. (A): Positive staining with pan-cytokeratin (CKAE1/3). (B) Positive staining (nuclear) with p63. (C): Positive staining with S100. (D): Positive staining with glial fibrillary acidic protein (GFAP).

Immunohistochemical staining revealed positive expression with pan-cytokeratin (CKAE1/AE3), anti-smooth muscle actin (ASMA), S100, p63, and glial fibrillary acidic protein (GFAP), as shown in Figure 3. The postoperative course of the patient was unremarkable. No further chemoradiation was given.

## Discussion

Myoepithelial carcinomas are rare salivary gland tumours that can occur at any anatomical location. We presented a case of myoepithelial carcinoma of the floor of the mouth with the involvement of major structures at the skull base. Grossly, myoepithelial carcinomas appear as unencapsulated infiltrative masses with areas of haemorrhage and cystic degeneration. Microscopically, they have solid, trabecular, reticular patterns or sheets of a spindle or plasmacytoid cells with a myxoid background. Immunohistochemistry for cytokeratin and myoepithelial markers (including S100, p63, GFAP, ASMA, vimentin, and calponin) is considered diagnostic.

The clinical behaviour of myoepithelial carcinoma is quite variable. It tends to recur locally and often metastasize. Hornick JL et al. showed that approximately 30% of the patients had metastatic disease, while 40% had local recurrence postoperatively [4]. The mainstay of treatment is wide surgical excision. Due to local recurrence, complete resection with disease-free margins is sufficient, which in turn depends upon the tumour size and infiltration of the surrounding structures. Hayashi A et al. reported a rare case of a parotid gland myoepithelial carcinoma measuring 40 cm in diameter without invasion of any major neurovascular or bony structures [5]. Similar cases of huge myoepithelial carcinomas have been reported [6,7]. Despite having large diameters, none of these cases had a locally aggressive disease or distant metastasis.

Large myoepithelial tumours have also been reported at sites other than head and neck. Hashmi et al. reported a case of a large mediastinal myoepithelioma adherent to parietal pleura and effacing the pulmonary parenchyma; however, no invasion of the adjacent soft tissues or bone was noted [8]. In our study, the size was less than those reported in the studies; however, the tumour was locally aggressive (encasing the internal carotid and involving the pterygoid muscle) and could not be completely excised with disease-free margin.

The differential diagnoses of myoepithelial carcinoma on histology include extra-skeletal myxoid chondrosarcoma, malignant melanoma, myxofibrosarcoma, and other myxoid soft tissue tumours. However, the distinction can easily be made by immunohistochemistry, as the expression of cytokeratins and myoepithelial markers like p63, ASMA, GFAP is not expressed in other tumours mimicking myoepithelial carcinoma morphologically [4]. The main diagnostic challenge in myoepithelial tumours is a differentiation between myoepithelioma and myoepithelial carcinoma, especially on truct/incisional biopsy, as in our case. The differentiation is based on an invasion into the surrounding tissue, moderate to severe atypia, with high mitotic count and necrosis presence [4].

Chromosome 8 aberrations are commonly seen in salivary gland myoepithelial carcinomas, as seen in other salivary gland malignant tumours [9]. In contrast, EWSR1, followed by FUS, are the commonly rearranged genes in soft tissue myoepithelial carcinoma [10,11]. We did not evaluate genetic alterations in our case, which is one of our study's limitations. Soft tissue myoepithelial tumours are distinct from the salivary gland myoepithelial tumours, as evidenced by molecular/genetic testing, however, many authors included myoepithelial tumours of the head and neck, outside major salivary glands (e.g. parotid), in soft tissue myoepithelial tumours [4]. Therefore, it is still a matter of debate whether to consider head and neck myoepithelial tumours in soft tissue myoepithelial tumours of salivary gland tumours. The head neck contains minor salivary glands. A lack of long-term follow-up is yet another limitation of our study.

## Conclusions

Myoepithelial neoplasms are rare tumours that can occur at any anatomical location. Histopathologically, these tumours can show diverse morphological patterns from epithelioid to spindle cells with myxoid to the hyalinized stroma. Therefore, immunohistochemical stains are necessary to reach a definitive diagnosis. Moreover, a distinction between a myoepithelioma and myoepithelial carcinoma is difficult on truct/incisional biopsy. The occurrence of myoepithelial carcinomas at complex anatomical locations, such as the floor of the mouth, is a diagnostic and therapeutic challenge, as due to large size and local aggressiveness, complete surgical excision is difficult.
